# The spectrum of intraoral bacteria seen in patients with cleft palates in an African setting

**DOI:** 10.1002/mbo3.679

**Published:** 2018-06-27

**Authors:** Shaal Ramdial, Anil Madaree

**Affiliations:** ^1^ Department of Plastic and Reconstructive Surgery Inkosi Albert Luthuli Central Hospital University of KwaZulu‐Natal Durban South Africa

**Keywords:** Africa, bacteria, cleft palate, palatoplasty

## Abstract

Dehiscence or palatal fistula formation following palatoplasty is a complication that has grave consequences for the patient that include tissue loss, emotional distress to the parents and patient, and further medical costs. Palatal dehiscence or fistula formation is multifactorial following surgery—tension of wound closure, poor patient adherence to postoperative orders and wound infection are the most common causes for this. Oral colonization with pathogenic organisms could play a role in wound healing complications. Identification of intraoral bacteria among patients with cleft palates has thus far not been performed. To identify the spectrum of intraoral bacteria in cleft palate patients in an African setting; a retrospective, chart review was performed at Inkosi Albert Luthuli Central Hospital—a quaternary hospital in Durban, South Africa. All patients with unrepaired cleft palates who underwent palatoplasty in 2015 were included. Fifty‐two patients were included. Preoperative throat/palatal swabs were taken prior to palatoplasty. The various bacteria cultured from the aforementioned swabs were cataloged. Various bacteria were cultured. In total, 23 patients (44.2%) had positive swab cultures. Eighteen cultures (34.6%) had gram‐positive growth only, four cultures (7.7%) had gram‐negative growth only, while one patient (1.9%) cultured both a gram‐positive and a gram‐negative organism. *Streptococcus viridans* was the most commonly cultured organism (19.2%) while beta‐hemolytic streptococci were cultured from only 4 swabs (7.7%). Our study cataloged the commonly occurring bacteria found in unrepaired cleft palate patients in Africa. Further research into the clinical significance of each bacteria is advised.

## 1 INTRODUCTION

Dehiscence or palatal fistula formation following palatoplasty is a complication that has grave consequences for the patient that include tissue loss, emotional distress to the parents and patient, and further medical costs (Cocco, Antonetti, Burns, Heggers, & Blackwell, 2010). Palatal dehiscence or fistula formation is multifactorial following surgery—tension of wound closure, poor patient adherence to postoperative orders and wound infection are the most common causes for this (Deshpande et al., 2014; Hupkens, Lauret, Dubelaar, Hartman, & Spauwen, 2007; Zhang et al., 2014). Oral colonization with pathogenic organisms could play a role in wound healing complications.

Patients in our setting are not part of a homogenous group and come from various racial, cultural, ethnic and socioeconomic backgrounds; it is possible that they harbor bacteria different from those identified in studies from other regions. In light of this, we sought to identify the spectrum of intraoral bacteria in our local cleft palate population.

## 2 MATERIALS AND METHODS

Inkosi Albert Luthuli Central Hospital (IALCH) in Durban, is a quaternary level hospital, which serves the province of Kwa‐Zulu Natal in South Africa. Among its various disciplines is the Department of Plastic and Reconstructive Surgery which, among other services, provides comprehensive cleft care to those in need. Patients with cleft palates are referred to the IALCH Plastic Surgery clinic from a variety of primary, district and regional healthcare facilities.

The vast majority of the patients underwent a palatoplasty at nine months of age unless they were not fit for surgery or were referred late to the department. Patients were not prescribed antibiotics or any special diets leading up to surgery, and were given an admission date a day prior their operation. Patients were kept nil per os on the day of the surgery and no intraoral mouthwashes were administered.

In theater, the patients were intubated under sterile conditions and no intraoral irrigation was used. Cleaning of the patient was performed with a povidone‐iodine solution and involved external surfaces only—no intraoral cleaning was performed. All operations were performed under sterile conditions with the surgeon, assistants and scrub sister in sterile gowns.

After the insertion of the Dingman retractor by the operating surgeon, an intraoral pus swab was taken with attention paid to the throat and palatal cleft. This pus swab was sent to the Microbiology laboratory for microscopy, culture and sensitivity testing. A prophylactic antibiotic (amoxicillin/clavulanic acid) was then administered intravenously. Palatoplasty was performed according to the departmental protocols. All patients were given oral analgesia and an antibiotic (amoxicillin/clavulanic acid) postoperatively with the antibiotic regimen changing, if needed, based on the pus swab results.

Patients with cleft palates, who had undergone a palatoplasty in 2015, had their charts reviewed. Results from the preoperative intraoral pus swabs were noted together with their age, gender and type of cleft palate. Cleft palates were divided into either an incomplete or complete group. Results were subsequently compared to similar studies performed locally and abroad.

Ethical approval for this study was granted by the Biomedical Research Ethics Committee (BREC) at the University of Kwa‐Zulu Natal (BREC reference number BE197/16). For this type of study formal consent is not required.

## 3 RESULTS

Fifty‐two patients underwent palatoplasty during the aforementioned timeframe. Thirty were male and 22 were female, with a male‐to‐female ratio of 1.36:1. The age range was between 8 months and 23 years. The majority of these patients (29 out of 52; 55.8%) were less than or equal to 18 months of age—the age below which palatoplasty is advised for optimal speech development (Goldstein et al., 2014; Ha et al., 2013). Twelve patients (23%) had complete cleft palates and 40 patients (77%) had incomplete cleft palates. (Table 1).

**Table 1:** Microbiology

	*n*	Percentage of cohort
Positive pus swabs	23	44.2%
Negative pus swabs	29	55.8%
Single organism cultured	20	38.5%
Multiple organisms cultured	3	5.8%
Swabs with gram‐positive organisms only	18	34.6%
Swabs with gram‐negative organisms only	4	7.7%
Swabs with gram‐positive AND gram‐negative organisms	1	1.9%

Twenty‐three patients (44.2%) cultured organisms (single or multiple) from their intraoral pus swabs. Only three of these patients (5.8% of the entire cohort) cultured more than one organism. Single organism growth was present in 20 swabs (38.5%). Eighteen cultures (34.6%) had gram‐positive growth only, four cultures (7.7%) had gram‐negative growth only, while one patient (1.9%) cultured both a gram‐positive and a gram‐negative organism. Twenty‐nine patients had clean (no growth) pus swabs (55.8%). (Table 2).

**Table 2:** Sample statistics

	*n*
Total patients	52
Male	30
Female	22
Complete cleft palate	12
Incomplete cleft palate	40

**Figure 1:**
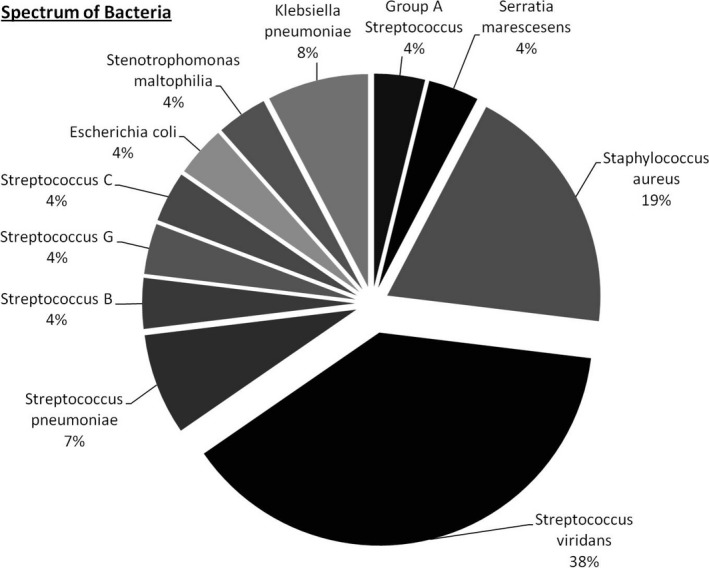
Spectrum of bacteria cultured

There were several organisms cultured from our cohort. Overall, the gram‐positive organisms cultured were *Group A streptococcus*,* Staphylococcus aureus*,* Streptococcus viridans*,* Streptococcus pneumoniae*,* Streptococcus B*,* Streptococcus G* and *Streptococcus C*. The gram‐negative organisms cultured were *Serratia marescesens, Escherichia coli*,* Stenotrophomonas maltophilia* and *Klebsiella pneumoniae*. (Figure 1).

Streptococci were the most common organisms cultured and found in 16 of the patients (30.8%). Of these, β‐hemolytic streptococci were identified in four patients (7.7% of all patients). The most commonly cultured streptococcal organism in our cohort was *Streptococcus viridans* (19.2% of all patients). The next most commonly cultured organism was *staphylococcus aureus* (5 patients; 9.6% of all patients).

Although not considered as bacteria, yeasts were cultured in two patients (3.9%).

Of the 23 patients who had positive bacterial cultures, nine (39.1%) went on to develop palatal fistulae. The cultured bacteria in these patients were *Streptococcus viridans*,* Staphylococcus aureus*,* Escherichia coli*,* Streptococcus pneumonia*,* Streptococcus group G* and *Stenotrophomonas maltophilia*. Gram‐negative bacteria were cultured in only two of the nine patients who developed palatal fistula.

## 4 DISCUSSION

The human oral cavity serves as an entry point to the digestive tract and is a veritable zoo of microbes. Approximately, 700 different bacterial species have been identified in this area (Wang, 2013). There has been limited research conducted in Africa in this field (Mÿburgh & Bütow, 2009; Roode, Bütow, & Naidoo, 2017).

As mentioned earlier, dehiscence or palatal fistula formation following palatoplasty is a complication that has far‐reaching consequences for the patient (Cocco et al., 2010). Palatal dehiscence or fistula formation is a multifactorial postoperative complication following surgery with wound infection being a major contributor (Deshpande et al., 2014; Hupkens et al., 2007; Zhang et al., 2014). Palatal surgery can be defined as a clean/contaminated procedure as the surgical wound is an entry portal through which the micro‐organisms invade. There is a strong relationship between preoperative cultures and postoperative palatal dehiscence, particularly *Group A Streptococcus* and *Staphylococcus aureus* (Hupkens et al., 2007).

Of particular interest to surgeons is the presence of β‐hemolytic streptococcus. Although all bacteria in levels greater than 10^5^ organisms per gram of tissue can cause clinical infection, only β‐hemolytic streptococci appear to be capable of routinely causing infection at levels of less than 10^5^ or 100,000 organisms per gram of tissue (Franz, Steed, & Robson, 2007).

Hupkens et al. (2007) sought to preoperatively identify bacteria that have a higher propensity to cause wound healing problems (*Group A Streptococcus* and *S. aureus*). Should the aforementioned bacteria be cultured, these patients were treated with intravenous amoxicillin‐clavulanate at certain times during their stay in the hospital. Of the 124 patients in their study, eight of them had positive cultures for *Group A Streptococcus* (for which seven were treated). The sole patient who did not receive antibiotics ultimately developed a palatal dehiscence. They went on to conclude that carriers of *Group A Streptococcus* were predisposed to wound infection. The most common species cultured in their cohort were *Hemophilus influenzae*,* Moraxella catarrhalis*,* Streptococcus pneumoniae* and *Staphylococcus aureus*.

A prospective study by Cocco et al. (2010) aimed to preoperatively identify the oral cavity flora in cleft palate patients. In their study, they identified *S*. *aureus* as being more prevalent in patients with cleft palates. They also concluded that screening for *Group A Streptococcus* should be performed prior to surgery, as its presence was associated with a three‐fold increase in palatal dehiscence. The most common organisms cultured in their cohort were *Klebsiella pneumoniae*,* Enterobacter cloacae*, and *Staphylococcus aureus*.

Adeyemo et al. (2013) looked at intraoperative blood cultures in patients undergoing cleft lip and palate surgery. As many patients with cleft palates have concurrent congenital heart disease, they wanted to assess intraoperative bacteraemia as a risk factor for bacterial endocarditis. Samples were taken just prior to intubation, one minute after placement of the final suture, and fifteen minutes after placement of the final suture. The most commonly cultured species were coagulase‐negative *Staphylococcus*,* Acinetobacter lwoffii*, and coagulase‐positive *Staphylococcus*. They advocate the use of broad‐spectrum prophylactic antibiotic cover for patients undergoing cleft lip or palate repair.

Mÿburgh & Bütow (2009), when assessing soft palate clefts alone, performed cultures on days 0, 2, 4 and 6. The most commonly cultured pathogenic organisms on day 0 were (in order of prevalence) *Staphylococcus aureus*,* Klebsiella pneumoniae*,* Candida albicans* and *Escherichia coli*. This profile gradually changed such that by day 6 the most commonly cultured organisms (in order of prevalence) were *Candida albicans*,* Pseudomonas aeruginosa* and *Enterobacter cloacae*. Three out of the four patients in this study who developed palatal dehiscence cultured *S. aureus*.

In their study assessing the value of preoperative microbiological screening of patients undergoing cleft palate repair, Thomas, Sibley, Goodacre, & Cadier (2012) stated that patients with unrepaired cleft lips and palates are at an increased risk of colonization with pathogenic organisms, in particular *Group A Streptococcus* and *S. aureus*, in comparison to the general population. Two hospitals were used in the study (Hospital A and B). Swabs were cultured 7–14 days prior to surgery and again on the operating table prior to commencement of surgery. Patients who cultured positive for *S. aureus* or *Group A Streptococcus* in Hospital A were treated with antibiotics and proceeded to surgery. Patients who cultured positive for *Group A Streptococcus* in Hospital B had their surgery deferred pending clear swabs. The most commonly cultured organism was *S*. *aureus* accounting for 21% of all cultures. *Group A Streptococcus* was found in only 3% of all patient cultures. They noted that wound dehiscence was significantly more likely in the presence of *Group A Streptococcus*. Unfortunately, in this group, identification of *Group A Streptococcus* was made only on the operative pus swab, with the preoperative pus swab failing to reveal this. They go on to conclude that preoperative swabs do not reliably predict the oropharyngeal flora at the time of surgery, and caution against its routine use.

Chuo & Timmons (2005) emphasized the detrimental effects postoperative wound infections can have in cleft lip and palate surgery—wound dehiscence, fistulae, poor speech, poor growth, poor esthetic result and even death. They felt that knowing which patients are carriers of pathogenic organisms would assist them in reducing the chance of postoperative infective complications. In their study, they identified *Staphylococcus aureus* and Beta‐hemolytic *Streptococcus* as being pathogenic.

Rennie, Treharne, & Richard (2009) echoed the belief that pathogenic bacteria in the oral cavity contribute to palatal dehiscence and fistula formation. In this study, preoperative throat swabs were taken, with the most commonly cultured organisms being *Group A Streptococcus* (25%) and *Staphylococcus aureus* (60%). The remaining 15% of throat swabs cultured other organisms, which were not named in the paper.

Studies have shown a higher incidence of dental caries in patients with oral clefts compared to noncleft controls (Sundell, Ullbro, Marcusson, & Twetman, 2015). These patients frequently have poor oral hygiene, enamel hypoplasia, and early colonization of caries‐inducing microorganisms. In their study, Sundell et al. (2015), collected saliva samples from patients with and without oral clefts. They noted that oral hygiene may be impaired in the cleft population owing to many factors—fear of brushing around the cleft area, the anatomy of the cleft itself, restricted access for tooth brushing and a higher incidence of misaligned teeth. They were unable to demonstrate an increase in *Streptococcus mutans*; however, the cleft group displayed higher levels of salivary lactobacilli. Although this study examined the role of bacteria in caries formation in the cleft population, it clearly demonstrates an alteration in the oral flora.

Our study demonstrated a wide variety of organisms in the oral cavity of a cleft palate patient. Both gram‐positive and gram‐negative organisms were cultured. The predominant organism cultured was *Streptococcus viridans*. This is not unexpected as *Streptococcus viridans* is the first coloniser of the human mouth, followed by *Staphylococcal*,* Veilonella*,* Neisseria* and other *Streptococcal* species in the first year of life. Furthermore, increasing one's sugar intake will facilitate the growth of more Streptococcus species. Older child would thus have more Streptococcal species as they are able to consume a wider variety of sugary foods (Arief, Mohamed, & Idris, 2005).

The second most common organism cultured in our study was *Staphylococcus aureus*. Its high prevalence in our population is in keeping with other studies (Adeyemo et al., 2013; Chuo & Timmons, 2005; Cocco et al., 2010; Hupkens et al., 2007; Mÿburgh & Bütow, 2009; Rennie et al., 2009; Thomas et al., 2012).

Our study results differ from many of the aforementioned studies in that a number of our patients cultured gram‐negative organisms. This could be that other studies did not test for these organisms, or the results were discarded, as they were not part of the study objectives. Nonetheless, the significance of gram‐negative organisms in our cohort is uncertain. Further evaluation into wound healing and postoperative complications among these patients is needed.

It is interesting to note that 39.1% of patients who cultured positive for intraoral bacteria ultimately developed a palatal fistula. Of these nine patients, only two had positive cultures for gram‐negative bacteria. Unfortunately, our study cohort was too small to draw any meaningful conclusions from this observation; however, it would appear that pathogenic intraoral bacteria could affect wound healing following palatoplasty. There appears to be no correlation between fistula development and the age of the patient. The organisms cultured were, similarly, unaffected by the age of the patient.

Three studies bear special mention: Adeyemo et al. (2013), Mÿburgh & Bütow (2009), and Roode et al. (2017) are studies from Africa and as such could be seen to address similar concerns to ours. There are subtle differences with the first two studies though. Adeyemo et al. focused on bacteremia following cleft palate surgery. This differs from our study, which looked at oral cavity colonization. Mÿburgh and Bütow only included patients with soft palate clefts whereas our study looked at both soft and hard palate clefts. Roode et al. looked at similar preoperative palatal swabs and their impact on antibiotic use. Their study highlighted the need for preoperative cultures in order to make appropriate antibiotic choices. Through our study, we have added to this body of knowledge.

Our study has limitations. The large number of patients (55.8%) who did not culture organisms from their pus swabs is of concern. This could be due to sampling errors—the swabs focused on the throat and palate. Perhaps more comprehensive swabbing of the oral cavity may yield more positive swabs. Another possibility is that the Microbiology laboratory may not report on oral commensals, which are cultured. Pus swabs themselves may have lower bacterial yields than tissue biopsies; however, we did not feel this was justified in light of the potential morbidity.

There is room for further study. Although β‐hemolytic streptococci have the potential to cause serious tissue injury, their role in cleft palate surgery remains uncertain. A link between these organisms and postoperative palatal dehiscence and fistula formation should be sought. Similarly, the role of gram‐negative bacteria should be investigated. The role of rapid detection systems for *Group A Streptococcus* has been validated (Gazzano et al., 2016; Hudson, Theron, Roditi, & Bloch, 1991; Wang et al., 2017) and we feel there could be a role for such kits in cleft palate surgery.

## 5 CONCLUSION

Our study has added to the body of knowledge concerning the spectrum of intraoral bacteria among cleft palate patients in an African setting. The significance of each organism will require further study. Identifying pathogenic organisms preoperatively could potentially lead to better cleft palate repair results.

## CONFLICT OF INTEREST

None declared.
